# Long read nanopore DNA sequencing with adaptive sampling to identify tyrosine kinase fusion genes

**DOI:** 10.1038/s41375-025-02801-5

**Published:** 2025-11-18

**Authors:** Matthew Salmon, Nicole Naumann, Jenny Rinke, Manja Meggendorfer, Deepti Radia, Mark Pomfret, Thomas Ernst, Andreas Hochhaus, Andreas Reiter, William J. Tapper, Helen White, Nicholas C. P. Cross

**Affiliations:** 1 Faculty of Medicine, University of Southampton, Southampton, UK; 2 Wessex Genomics Laboratory Service, Salisbury, UK; 3 Department of Hematology and Oncology, University Hospital Mannheim, Heidelberg University, Mannheim, Germany; 4 Klinik für Innere Medizin II, Universitätsklinikum Jena, Jena, Germany; 5 MLL Munich Leukemia Laboratory, Max-Lebsche-Platz 31, Munich, Germany; 6 Department of Clinical Haematology, Guy’s and St Thomas’ NHS Hospitals, London, UK; 7 All Wales Genomics Laboratory, Cardiff, UK

**Keywords:** Genomics, Cancer genomics

## Abstract

Diverse haematological neoplasms are driven by tyrosine kinase (TK) fusion genes formed by recurrent or non-recurrent genomic rearrangements. The resulting chimeric proteins often present excellent targets for treatment with kinase inhibitors, and the fusion transcripts or genomic junctions can be used as specific targets for molecular monitoring. Whilst the TK genes involved are generally well characterised (e.g. *ABL1, PDGFRA, FGFR1*), the fusion partners are very diverse, presenting a challenge for detection and characterisation of these structural variants (SV) using current diagnostic methods. We assessed the ability of targeted nanopore sequencing using adaptive sampling to detect fusion genes in myeloid neoplasms. We sequenced genomic DNA from patients (*n* = 20) with a known or suspected TK gene fusion and identified rearrangements in 18 cases, including all cases with a known TK fusion, typical and atypical *BCR::ABL1* rearrangements, an 843Kb deletion causing a *FIP1L1::PDGFRA* fusion, novel *AGAP2::PDGFRB* and *NFIA::PDGFRB* fusions, and a complex *CCDC88C::PDGFRB* rearrangement with multiple translocation events. The approach was fast (<72 h/sample from DNA to result), flexible with minimal hands-on laboratory time, and provided accurate, patient-specific characterisation of genomic breakpoints.

## Introduction

Structural variants (SV) that give rise to tyrosine kinase (TK) fusion genes are seen recurrently in myeloid neoplasms, specifically chronic myeloid leukaemia (CML), which is defined by the presence of *BCR::ABL1*, but also in the much less common entity “myeloid/lymphoid neoplasms with eosinophilia and tyrosine kinase gene fusions” (MLN-TK), characterized by rearrangements of *PDGFRA*, *PDGFRB*, *FGFR1*, *JAK2*, *FLT3*, *ETV6::ABL1* and other TK fusions [1, 2]. TK fusion proteins are excellent treatment targets for tyrosine kinase inhibitor (TKI) therapy as well as serving as sensitive markers for assessment of measurable residual disease (MRD) [3–13]. Consequently, accurate detection of TK fusions is critical to effective diagnosis and management.

For CML, detection of *BCR::ABL1* is relatively straightforward, although around 2% of cases have an atypical variant that may be missed during diagnostic workup [14, 15]. For MLN-TK, however, the situation is much more complex. Not only are there multiple TK genes that drive the disease, but each TK has multiple partner genes with nearly 100 different partner gene/TK fusions described to date [13]. Most are associated with visible karyotypic rearrangements, but a growing number are cytogenetically cryptic. Gene-level diversity is further compounded by heterogeneity of genomic breakpoints that lead to different exon usage. Whilst the breakpoints in some TKs are tightly clustered, such as the *PDGFRA* exon 12 breakpoint region [16, 17], others can be spread over large distances, such as the *ABL1* breakpoint region in CML, which spans around 140 Mb [18]. Some fusions, e.g. *PCM1::JAK2*, show marked variation in mRNA fusion junctions between patients [19].

This diversity poses challenges for the comprehensive detection of fusion genes. At the DNA level, targeted approaches would require very high levels of multiplexing and would be unable to detect rearrangements involving novel partners or atypical breakpoints. Short-read whole genome sequencing (WGS) is effective in many cases but has general difficulties in detecting SVs, with one study estimating that up to 70% of SVs may be missed [20]. At the RNA level, whole transcriptome sequencing or targeted one sided transcriptome sequencing approaches can be effective, but these methods (along with WGS) require batching of large numbers of samples to be economical.

Long-read sequencing is emerging as a superior method for the detection and full characterization of SVs compared to short-read NGS [20, 21] as the length of sequence reads (on the scale of several Kb and above) will often contain the entire rearrangement and sufficient flanking sequence to enable unambiguous identification. Additionally, nanopore sequencing offers the ability for computational, PCR-free target enrichment by adaptive sampling, whereby a list of regions of interest (ROI) are provided to the sequencer, against which each DNA fragment is checked in real-time as sequencing progresses. If a fragment can be mapped to the ROI it is sequenced in full, but if the strand contains non-ROI sequence it is ejected from the pore, allowing a new strand to enter, and so maximising the sequencing time of DNA with ROI sequence [22, 23].

To assess the ability of long read sequencing to detect and characterise DNA rearrangements, we designed a targeted long-read nanopore sequencing panel using adaptive sampling to detect genomic fusion events involving TK and other genes recurrently involved in haematological malignancies. We demonstrate that this method is an effective way to detect and characterize TK fusions in CML and MLN-TK.

## Methods

### Patient samples

Samples from patients with CML, MLN-TK or related disorders were sourced from the Wessex Genomics Laboratory Service, Salisbury (*n* = 9), University Hospital Jena (*n* = 7) or University Hospital Mannheim (*n* = 4). Samples were selected to cover a diversity of common TK rearrangements (in cases where the fusion was already known), as well as to aid the characterisation of the rearrangement in cases where the precise SV was unclear, but a TK fusion had been implicated by clinical phenotype, cytogenetics, FISH or short-read NGS. (Table 1). Samples were not blinded; the results from prior testing, including the identities of the genes known or suspected to be involved, were available during the analysis. DNA from peripheral blood or bone marrow leucocytes was extracted using routine methods, according to local protocols. Informed consent was obtained from all patients. The study design adhered to the tenets of the Declaration of Helsinki and was approved by the National Research Ethics Service (UK) Committee South West, the institutional ethics committee of the University Hospital Jena, and the institutional review board of the Medical Faculty of Mannheim, Heidelberg University as part of the ‘German Registry on Disorders of Eosinophils and Mast Cells’.

**Table 1:** Summary of patient details at time of referral.

Sample	Working diagnosis/ referral reason	Routine genetic results	Known/suspected Fusion
S_1	CML	46,XY,t(9;22)(q34;q11.2),der(9)t(9;14)(q?34;q?32),add(9)(q?34),add(14)(q?32), der(22)t(1;22)(p?36.1;q?13) [21]	*BCR::ABL1* e14a2
S_2	Ph+ ALL	52~55,XY,+4[15],+6[19],der(9)t(9;22)(q34;q11.2)[20],+13[15],+14[4],+15[8],+der(22)t(9;22)[5] or +der(22)t(9;22)x2[12],+1~6mar[cp20]	*BCR::ABL1* e1a2
S_3	CML	N/A	*BCR::ABL1* e1a2
S_4	CML	46,XX,t(9;22)	*BCR::ABL1* e14a3
S_5	CML	t(9;22;20)(q34;q11.2;q11.2)	*BCR::ABL1* e1a2
S_6	CML	46,XY,t(9;22)	*BCR::ABL1* e13a2
S_7	MLN-TK	Fusion detected by FISH, confirmed by RT-PCR	*FIP1L1::PDGFRA*
S_8	MLN-TK	t(8;22)(p11;q11). Fusion confirmed by RT-PCR	*BCR::FGFR1*
S_9	MLN-TK	Fusion confirmed by RT-PCR	*BCR::JAK2*
S_10	MLN-TK	Susptected *ETV6::ABL1* from SNP array. Inconclusive cytogenetics. FISH negative for *BCR::ABL1, FIP1L1::PDGFRA, PDGFRB* or *FGFR1* rearrangements.	*ETV6::ABL1*
S_11	MLN-TK	*FIP1L1::PDGFRA* negative. t(8;9)(p22;p24); RT-PCR detection of *PCM1::JAK2*	*PCM1::JAK2*
S_12	MLN-TK	46,XX,?t(6;10)(p21;q22),add(21)(q22). *BCR::ABL1, ETV6::PDGFRB* and *FIP1L1::PDGFRA* negative. Clinical response to trial of imatinib. Suspected *PDGFRB* rearrangement by WGS and RNAseq with possibly additional complexity. *CCDC88C::PDGFRB* confimed by RT-PCR	*CCDC88C::PDGFRB*
S_13	?MLN-TK	MPN with abnormal karyotype 46,XY,t(5;17)(q32;p11.2). *PDGFRB* rearangement detected by FISH. Poor TKI response. NGS myeloid panel negative.	*?17::PDGFRB*
S_14	?MLN-TK	t(5;12)(q32;q13). *PDGFRB* rearrangement with unknown partner detected by FISH *FIP1L1::PDGFRA*, *BCR::ABL1*, and *ETV6::PDGFRB* negative by RT-PCR. Clinical response to imatinib	*?::PDGFRB*
S_15	?MLN-TK	t(5;12)(q33;q24); *PDGFRB* split by FISH	*?12q24::PDGFRB*
S_16	?MLN-TK	46,XY,t(1;5)(p22;q32). *PDGFRB* rearrangement by FISH. *ETV6::PDGFRB* not detected by RT-PCR. Clincal response to imatinib.	*?::PDGFRB*
S_17	?MLN-TK	46,XX,t(4;12)(q12;p13). Suspected *ETV6::PDGFRA* detected by FISH. Failed to confirm rearrangment in DNA or cDNA. Overexpression of *PDGFRA* detected.	*?ETV6::PDGFRA*
S_18	?MLN-TK	46,XX[25]. WGS showed inversion of 4q, involving *PRKG2* and *PDGFRA*.	*?PKRG2::PDGFRA*
S_19	HES/?MLN-TK	Normal karyotype; NGS panel, RNAseq and WGS negative. Eosinophilia responded to treatment with imatinib.	None
S_20	CML	MRD sample, *BCR::ABL1* negative	*BCR::ABL1* negative

*CML* chronic myeloid leukaemia, *Ph+ ALL* Philadelphia chromosome positive Acute Lymphoblastic Leukaemia, *MLN-TK* myeloid/lymphoid neoplasms with eosinophilia and Tyrosine Kinase gene fusions, *HES* hypereosinophilic syndrome.

### Nanopore sequencing

Prior to sequencing library preparation, samples were fragmented using g-TUBEs (Covaris, Woburn, MA, USA) to a target size of 5–10 kb, according to the manufacturer’s instructions. Sequencing libraries were prepared using the Ligation Sequencing Kit V14 (Oxford Nanopore Technologies, Oxford, UK) according to the kit protocol. Sequencing was performed on MinION R10.4.1 flow cells using a MinION Mk1b instrument for 24 h, followed by a flow cell wash, reload, and sequencing for a further 24 h.

### Target panel design

Since adaptive sampling generally requires targeting of a substantial proportion of the genome to be effective, we selected an extended panel of 240 genes recurrently involved in CML, MLN-TK and other haematologic malignancies (Supplementary Table 1). Whole gene sequences between the transcription start and end (txStart/txEnd) coordinates of the Matched Annotation from NCBI and EMBL-EBI (MANE) transcripts in the UCSC genome browser database [24] were targeted. A buffer sequence of 11 kb was added to the 3’ and 5’ ends of each gene. See supplementary methods for further details.

### Basecalling and analysis

**Fig. 1:**
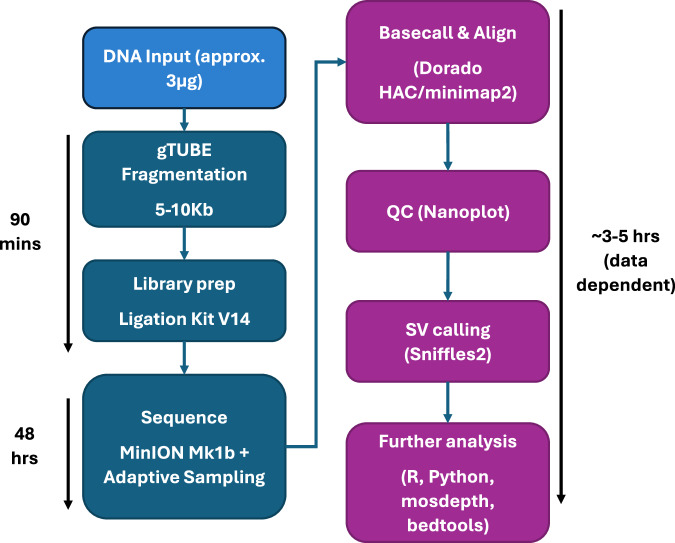
Sequencing workflow. Full sequencing workflow showing laboratory (dark blue) and analytical (purple) steps.

After nanopore sequencing, all samples were analysed with a common pipeline (Fig. 1). Briefly, basecalling was performed with the Dorado basecaller from Oxford Nanopore Technologies (ONT, version 0.52), using the HAC v4.3.0 model. Alignment to the hg38 human reference was performed with Minimap2 (as bundled with Dorado) and SVs were called with Sniffles2 [25] using the *--non-germline* option and all other parameters left as default (see Supplementary Methods for further details). Called SVs were annotated and prioritised with SvAnna [26], using default parameters and the Human Phenotype Ontology term for myeloproliferative disorders (HP:0005547) followed by manual curation of prioritised SV calls. Copy number alterations were called with QDNAseq [27] with a bin size of 15 kbp.

### Validation

PCR assays were designed to confirm predicted fusion junctions in genomic DNA based on nanopore results, and predicted fusion transcripts were targeted from cDNA where material was available. Amplicons were read by Sanger sequencing (Supplementary Table 2).

## Results

### Sequencing metrics

**Fig. 2:**
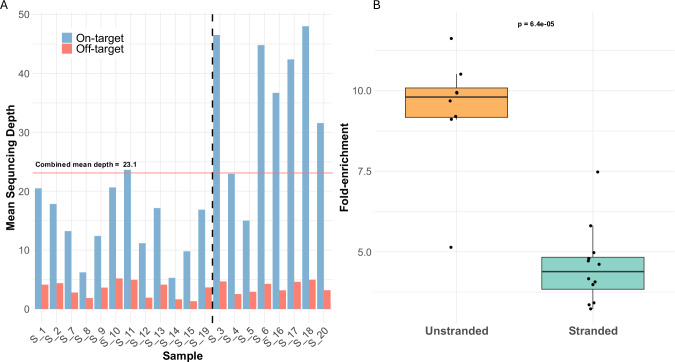
Sample sequencing depth and enrichment by adaptive sampling. **A** Per sample mean sequencing depth for on- (blue bars) and off-target (red bars) regions. Samples to the left of the dashed line had strand information specified in the BED file, samples to the right of the line did not. **B** Fold-enrichment of samples where strand information was omitted (unstranded) vs included (stranded) from the BED file. Wilcoxon rank sum test.

After filtering reads with Q-score < 8, a mean sequencing depth of 23.1x was achieved across the 240 genes targeted by adaptive sampling (on-target), compared to 3.5x for genomic regions not specified in the BED file (off-target). This represents an average 6.6-fold enrichment (Fig. 2A). A median of 19.02 million total reads were sequenced per-sample, of which 92,504 (0.74%) were classified as on-target, showing good concordance with the size of the adaptive sampling panel relative to the genome (0.74% on target reads vs 0.76% of the genome targeted). An overview of key metrics is shown in Table 2. The threshold for calling a fusion was automatically determined per-sample by Sniffles2 (Supplementary Methods) and the median number of reads supporting each fusion was 6 (range 2–59, Table 3).

**Table 2:** Summary of key sequencing metrics.

Median total reads/sample	19.02 M
Median bases/sample	11.8 Gb
Median on-target reads/sample	92 504
Median sequence length (total)	622 bp
Median sequence length (on-target)	4999 bp
Median on-target N50	7294 bp
Mean On-target depth	23.1x
Mean Off-target depth	3.5x
Mean overall target fold-enrichment	6.6
Mean On-target depth (Stranded)	14.6x
Mean Off-target depth (Stranded)	3.30x
Mean target-fold enrichment (Stranded)	4.55
Mean On-target depth (Unstranded)	36x
Mean Off-target depth (Unstranded)	3.79
Mean target-fold enrichment (Unstranded)	9.39

Stranded/Unstranded indicates inclusion/omission of strand information in the adaptive sampling BED file.

**Table 3:** Details of fusions detected by Nanopore sequencing.

Sample	Fusion Detected by Nanopore	SV Type	Partner (Non-TK) Gene Targeted	TK fusion breakpoint coordinates (hg38)	Additional findings	Fusion Supporting Reads	Strand specified in BED file
S_1	*BCR::ABL1* e14a2	Translocation	Y	chr9:130,723,854, chr22:23,292,107	t(9;14)(q34.12;q32.31)	11	Y
S_2	*BCR::ABL1* e1a2	Translocation	Y	chr9:130,806,756, chr22:23,246,666	–	13	Y
S_3	*BCR::ABL1* e1a2	Translocation	Y	chr9:130,806,754, chr22:23,238,110	–	50	N
S_4	*BCR::ABL1* e14a3	Translocation	Y	chr9:130,854,656, chr22:23,292,465	–	5	N
S_5	*BCR::ABL1* e1a2	Translocation	Y	chr9:130,826,244, chr22:23,204,452	t(20;22)(q11.22;q11.23), ?t(9;22)	2	N
S_6	*BCR::ABL1* e13a2	Translocation	Y	chr9:130,807,816, chr22:23,289,949	t(3;22)(p21.31;q11.23), additional t(9;22) breaks chr9:130,807,184, chr22:23,327,318	13	N
S_7	*FIP1L1::PDGFRA*	Deletion	Y	chr4:53,431,452, chr4:54,274,913	–	6	Y
S_8	*BCR::FGFR1*	Translocation	Y	chr8:38,416,802, chr22:23,266,238	–	2	Y
S_9	*BCR::JAK2*	Translocation	Y	chr9:5,079,992, chr22:23,218,500	–	4	Y
S_10	*ETV6::ABL1*	Translocation	Y	chr9:130,742,894, chr12:11,883,840	–	6	Y
S_11	*PCM1::JAK2*	Translocation	N	chr8:18,021,830, chr9:5,059,124	–	2	Y
S_12	*CCDC88C::PDGFRB* t(5;6;14)	Translocation (complex)	Y*	chr5:150,129,557, chr14:91,324,655	t(5;6)(q32;p21.1), t(6;14)(p24.1;q32.11), *CCDC88C* del (chr14:91,307,523-91,324,086)	7	Y
S_13	*MPRIP::PDGFRB*	Translocation	N	chr5:150,126,093, chr17:17,177,189	–	2	Y
S_14	*AGAP2::PDGFRB*	Translocation	N	chr5:150,127,064, chr12:57,727,583	–	3	Y
S_15	*SART3::PDGFRB*	Translocation	N	chr5:150,128,653, chr12:108,527,296	–	5	Y
S_16	*NFIA::PDGFRB*	Translocation	N	chr5:150,128,554, chr1:61,365,209	–	20	N
S_17	t(4;12)(q12;p13.2)	Translocation	N	chr4:54,079,924, chr12:11,691,419	–	24	N
S_18	*PKRG2::PDGFRA*	Inversion	Y*	chr4:54,274,934, chr4:81,172,224	–	59	N
S_19	Unknown	–	–	–	–	-	N
S_20	*BCR::ABL1* negative	–	–	–	–	-	Y

*Relevant partner gene was added to the individual BED file for these samples only.

**Fig. 3:**
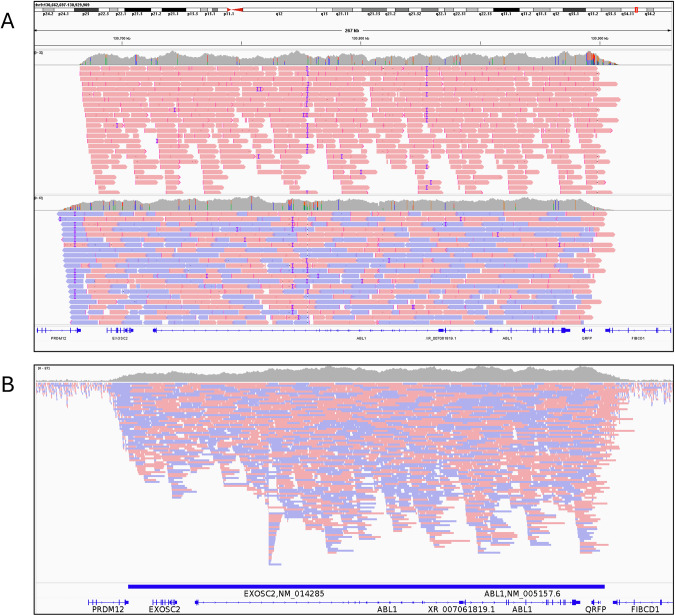
Stranded and unstranded reads. **A** Comparison of reads mapping to *ABL1* where a stranded (top track, S_5) or unstranded (bottom track, S_20) BED file was used. Red bars show reads on the + strand, blue bars the – strand. *ABL1* is located on the + strand. **B** Visualisation of adaptive sampling in S_3 (*BCR::ABL1*). Horizontal blue bar indicates the region targeted for adaptive sampling, and the corresponding increased depth of sequencing in this region, compared to adjacent untargeted regions.

During initial analyses, we observed that only reads mapping to either the + or – strand were being accepted, which corresponded to the strand of the target specified in the adaptive sampling BED file. Strand information was removed for subsequent sequencing runs (Table 3), and we observed significantly increased target enrichment (stranded mean enrichment = 4.55; unstranded mean = 9.39. Wilcoxon *p* < 0.0001, Fig. 2B) and depth of on-target sequencing (stranded mean on-target depth = 14.6x, unstranded mean = 36x. Wilcoxon *p* = 0.00071, Supplementary Fig. 1) of these samples. After excluding samples that had additional regions targeted (S_12, S_18, see below), the number of on-target reads approximately doubled when no strand was specified in the target file (stranded mean on-target reads = 78,384; unstranded mean = 143,418. Wilcoxon *p* = 0.0012, Supplementary Fig. 1), corresponding to the expected rejection of half the total DNA fragments (i.e. those with sequence mapping to the opposite strand) when the strand is specified (Fig. 3).

### Confirmation of known fusions and additional rearrangements detected by nanopore sequencing

Of the 20 patient samples tested, 12 had a known TK fusion that had previously been characterized by routine diagnostic tests (patients S_1–S_12). Nanopore sequencing detected the expected fusion in all 12 cases (Table 3), including common and atypical isoforms of *BCR::ABL1* (*n* = 6) and fusions in MLN-TK involving *JAK2* (*n *= 2), *ABL1, FGFR1, PDGFRA* or *PDGFRB* (*n* = 1 of each). An additional CML case tested negative for *BCR::ABL1* mRNA by RT-qPCR whilst on TKI therapy (S_20). As expected, nanopore sequencing did not detect a *BCR::ABL1* fusion. Overall, therefore, 13/13 cases were concordant between routine diagnostic analysis and nanopore sequencing.

Of the 6 *BCR::ABL1* positive cases, additional SV were detected in patients S_1, S_5 and S_6. Prior cytogenetic analysis of S_1 showed a karyotype of 46,XY,t(9;22)(q34;q11.2),der(9)t(9;14)(q?34;q?32),add(9)(q?34),add(14)(q?32), der(22)t(1;22)(p?36.1;q?13), and in concordance with this, nanopore sequencing detected a t(9;14)(q34.12;q32.31) in addition to the t(9;22) *BCR::ABL1* rearrangement. We did not detect the t(1;22) that was seen with cytogenetic analysis, which suggests the breakpoints for the t(1;22) could be outside of the regions we targeted with adaptive sampling. S_5 showed a t(9;22;20)(q34;q11.2;q11.22) by cytogenetics which was also detected by nanopore sequencing, with 2 breakpoints on chr20 within a 41 bp window at chr20:34,353,923 and chr20:34,353,964 (Table 3). These breaks were spanned by split reads aligning to *BCR* and *ABL1* respectively, suggesting the occurrence of 3 translocation events (Supplementary Fig. 2). We confirmed the t(20;22)(q11.22;q11.23) by PCR and Sanger sequencing (Supplementary Table 2), however the t(9;20) was unconfirmed, possibly due to an enrichment of Alu repeats in the vicinity of the chr20 breakpoints. S_6 had no additional SV detected by cytogenetics, however nanopore sequencing identified a t(3;22)(p21.31;q11.23), which was confirmed by Sanger sequencing (Supplementary Table 2). The chr22 breakpoint was in intron 13 of *BCR* (chr22: 23,289,852), 103 bp upstream of the *BCR::ABL1* breakpoint (Supplementary Fig. 3). The chr3 break was in intron 3 of *RBM6* at chr3: 49,999,659. *RBM6* and *BCR* are in the same genomic orientation, leading to the potential for generation of a *RBM6::BCR* fusion mRNA, however cDNA from this patient was unavailable to assess this possibility.

**Fig. 4:**
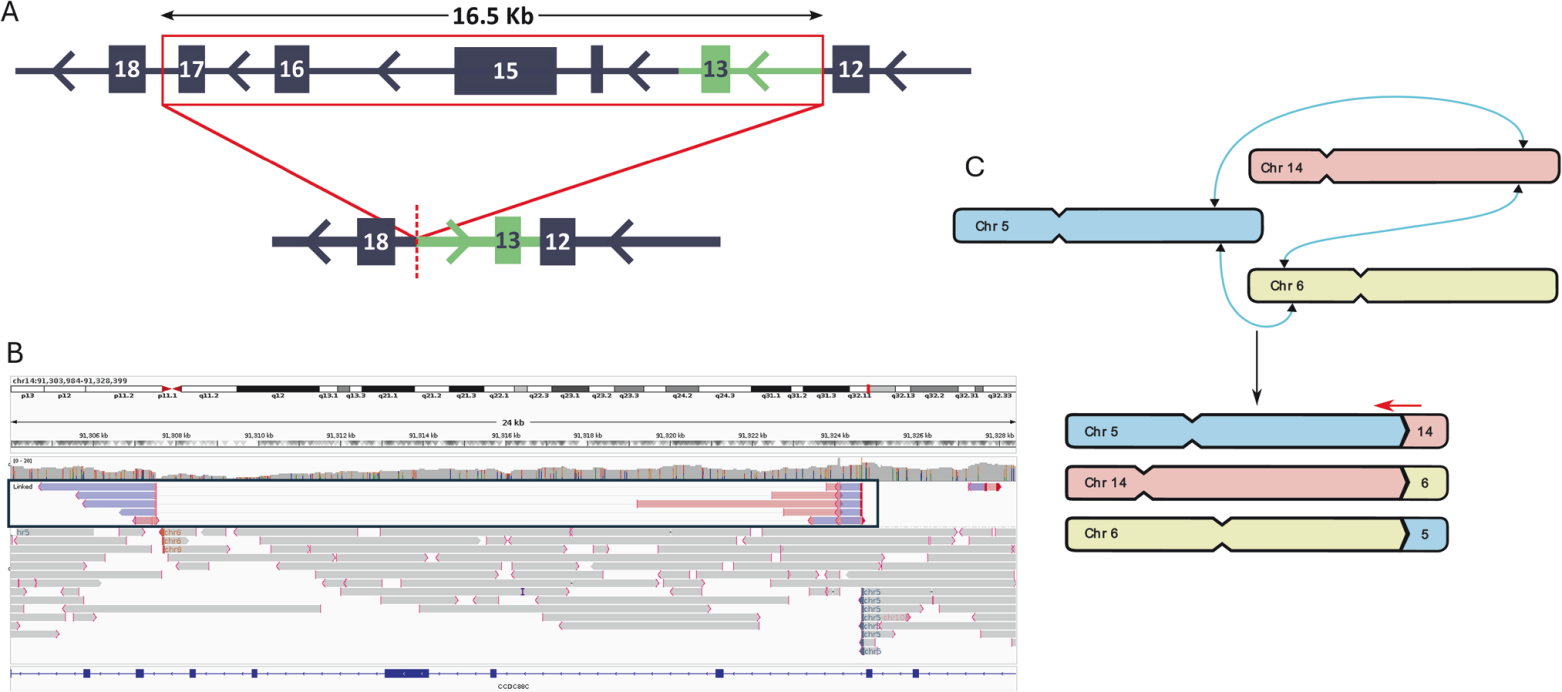
A complex rearrangement leading to a *CCDC88C::PDGFRB* fusion. **A** Schematic of the SV affecting *CCDC88C* S_11 with the deleted region indicated by the red box. Green section shows the inverted region that is inserted 5’ of the deletion breakpoint (bottom panel). Numbered boxes show *CCDC88C* exons. Arrows indicate genomic orientation. **B** Sequence reads spanning the SV visualised in IGV. Blue and pink linked reads (boxed) span the deletion/inversion/duplication. Grey reads spanning translocation breakpoint are labelled with the partner chromosome for t(5;14) and t(6;14). **C** Diagram of chromosomal rearrangements and approximate breakpoint locations (Blue lines). Red arrow indicates the pathogenic *CCDC88C::PDGFRB* rearrangement. Not drawn to scale.

Of the 6 MLN-TK cases, S_12 had a 46,XX,?t(6;10)(p21;q22),add(21)(q22) by cytogenetics, but other investigations (FISH, WGS, RNAseq, RT-PCR) revealed *CCDC88C::PDGFRB* as the most plausible driver event, suggesting a cryptic t(5;14)(q32;q32). We added *CCDC88C* to the adaptive sampling target BED file for this sample to increase the sequencing depth across the potential fusion partner, and nanopore sequencing revealed a complex translocation event involving concurrent t(5;14)(q32;q32.11), t(6;14)(p24.1;q32.11) and t(5;6)(q32;p21.1) (Fig. 4). The breakpoints on chr5 and chr6 for each rearrangement were close together, separated by 118 bp and 558 bp respectively. However, on chr14 (*CCDC88C*) the breakpoints were separated by a complex deletion/inversion event, where a 16.5 kb deletion is followed by a duplication of inverted sequence from the 3’ end of the deleted region (Fig. 4A). The inverted sequence was not fully characterized but was at least 5 kb. PCR and Sanger sequencing confirmed the genomic chr14 deletion event as well as the t(5;6) and t(6;14) (Supplementary Table 2). The breakpoint region on chr6 was intergenic, therefore the t(5;6) and t(6;14) would not be predicted to generate a fusion transcript.

S_10 had an *ETV6::ABL1* rearrangement confirmed by RT-PCR after multiple investigations, including inconclusive cytogenetics, FISH, and SNP-array (Table 1). Nanopore sequencing confirmed the genomic fusion with breakpoints in *ETV6* intron 5 and *ABL1* intron 1 (Table 3). In concordance with previous reports [28], RT-PCR and Sanger sequencing revealed the co-expression of two distinct fusion transcripts types, with *ETV6* exon 4 (type A) or exon 5 (type B) joined to *ABL1* exon 2 (Supplementary Table 2).

### Identification of previously uncharacterised TK fusions

Samples S_13 - S_19 were from patients with a clinical phenotype suggestive of MLN-TK but a specific TK fusion had not been identified by standard diagnostic methods. Nanopore sequencing was able to characterise a TK rearrangement in 6/7 of these cases. Patient S_13 had a 46,XY,t(5;17)(q32;p11.2), and FISH indicated a rearrangement of *PDGFRB* with an unknown partner. Targeted NGS myeloid panel sequencing did not detect any known actionable variants. Nanopore sequencing confirmed a rearrangement of *PDGFRB* and identified the partner gene as *MPRIP* at 17p11.2, with breaks in *MPRIP* intron 21 and *PDGFRB* intron 11 (Table 3). Sanger sequencing confirmed the presence of the genomic fusion, which would suggest a fusion transcript with junctions between *MPRIP* exon 20 and *PDGFRB* exon 12 (Supplementary Tables 2 and 3), however no RNA sample was available for further confirmation.

**Fig. 5:**
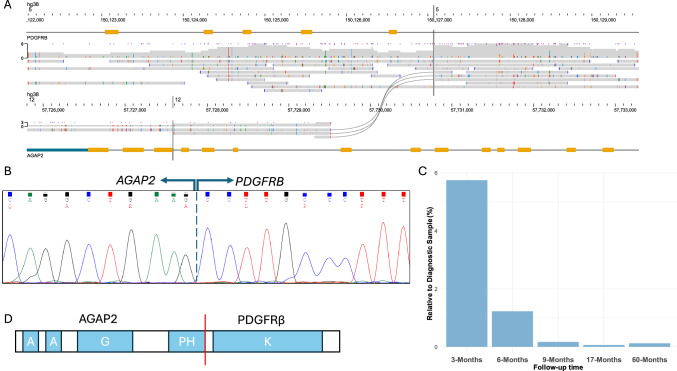
Characterization of a novel *AGAP2::PDGFRB* fusion. **A** Nanopore sequencing across *AGAP2::PDGFRB*. Black splines indicate fusion spanning reads. **B** Sanger sequencing of *AGAP2::PDGFRB* cDNA transcript showing the fusion junction (dashed line) between *AGAP2* exon 13 and *PDGFRB* exon 11 . **C** RT-qPCR analysis of follow up samples showing reduction of fusion transcript levels relative to the diagnostic sample. **D** Schematic of predicted fusion protein structure. A: Ankyrin repeats, G: GTPase-activating protein (GAP) domain, PH: Pleckstrin homology, K: protein kinase domain. Not drawn to scale.

S_14 was from a patient presenting with suspected MLN-TK and a t(5;12)(q32;q13) and involvement of *PDGFRB* by split-apart FISH. A trial of imatinib had produced a clinical response, however tests for *FIP1L1::PDGFRA* and *ETV6::PDGFRB* were negative (Table 1). Nanopore sequencing identified a genomic *AGAP2::PDGFRB* fusion, with genomic breaks in *AGAP2* intron 13 and *PDGFRB* intron 10 (Table 3, Fig. 5). The presence of a fusion transcript with a junction between *AGAP2* exon 13 and *PDGFRB* exon 11 was confirmed by RT-PCR and Sanger sequencing (Supplementary Tables 2 and 3) which then enabled testing of follow up samples by RT-qPCR (Supplementary Methods). After 9 months, fusion transcript expression had decreased to 0.17% of diagnostic levels, and remained below this level in 17- and 60-month follow up samples, indicating the fusion is sensitive to TKI treatment with imatinib in line with other *PDGFRB* rearrangements (Fig. 5C). To our knowledge, *AGAP2* at 12q14 has not been previously described as a fusion in MLN-TK or other haematological malignancies.

Patient S_15 had been previously found to carry a t(5;12)(q33;q24) involving *PDGFRB* as determined by FISH, but the partner gene was unconfirmed. Nanopore sequencing showed a breakpoint at chr12:108,527,296 within intron 15 of *SART3*, a known partner of *PDGFRB*, and the presence of a *SART3::PDGFRB* transcript was confirmed by RT-PCR, with the junction between exons 15 and 11 of *SART3* and *PDGFRB* respectively (Supplementary Tables 2 and 3). The *SART3* locus is at 12q23.3, very close (approximately 77Kb) to the 12q23.3/12q24.11 boundary at chr12:108,6000,000, highlighting the benefits of increased resolution afforded by our method over traditional cytogenetic techniques.

Patient S_16 was also suspected of carrying a *PDGFRB* fusion, with a 46,XY,t(1;5)(p22;q32) identified by cytogenetics, *PDGFRB* involvement by FISH and a clinical response to imatinib but no confirmed partner gene (Table 1). Nanopore sequencing detected a t(1;5)(p31.1;q32) leading to an *NFIA::PDGFRB* fusion (Table 3), and the fusion transcript joining exon 6 of *NFIA* to exon 11 of *PDGFRB* was confirmed by RT-PCR and Sanger sequencing (Supplementary Tables 2 and 3). This fusion has been previously described in AML [29], however this is the first time it has been detected in MLN-TK.

Patient S_17 had an 46,XX,t(4;12)(q12;p13) by cytogenetics suggesting the possibility of an underlying *ETV6::PDGFRA* fusion, however this was not detected by RT-PCR. Overexpression of *PDGFRA*, however, was detected using RT-qPCR [30]. Nanopore sequencing detected a t(4;12)(q12;p13.2), with the chr12 breakpoint in *ETV6*, however the chr4 breakpoint was at chr4: 54,079,923, an intergenic region approximately 149Kb upstream of *PDGFRA*. The closest gene to the chr4 breakpoint is *CHIC2*, however we were not able to detect an *ETV6::CHIC2* fusion transcript by RT-PCR. The genomic fusion was confirmed by PCR and Sanger sequencing (Supplementary Table 2).

Patient S_18 was cytogenetically normal (46,XX [25]), however short-read WGS had detected an inversion between *PDGFRA* and *PRKG2*, although no fusion transcript had been identified. Therefore, the entire 26.9 Mb separating the two genes was additionally targeted by nanopore sequencing to confirm the WGS result. The increase in total target size did not appear to have a detrimental effect on sequencing performance, with a mean on-target sequencing depth of 48x, the highest amongst all samples (Fig. 2). The SV was confirmed, with breaks in *PRKG2* intron 3 and *PDGFRA* exon 12. Targeted RT-PCR and Sanger sequencing confirmed the presence of an in-frame fusion transcript joining exon 3 of *PRKG2* to exon 12 of *PDGFRA*, with 6 bp of retained *PRKG2* intronic sequence at the fusion junction (Supplementary Tables 2 and 3).

No TK rearrangement was detected in patient S_19. This sample was taken from a patient with hypereosinophilic syndrome (HES) and normal karyotype that showed a haematologic response to treatment with imatinib. No evidence of a TK fusion or evidence for clonality was found by nanopore sequencing, or by routine investigations by targeted NGS, short-read WGS or RNA sequencing.

### Copy number alterations

As well as long on-target reads, the output of adaptive sampling experiments also contains the shorter (400–500 bp) reads that were rejected as being off-target and are spread across the genome, effectively providing a low-coverage whole genome sequence which can be used to interrogate copy number alterations (CNAs). To assess this in our samples, we initially analysed sample S_2, taken from a patient with p190/e1a2 Philadelphia chromosome positive (Ph + ) acute lymphoblastic leukaemia (ALL) and hyperdiploidy. Conventional cytogenetics at diagnosis showed a complex karyotype of 52~55,XY,+4[15],+6[19],der(9)t(9;22)(q34;q11.2)[20],+13[15],+14[4],+15[8],+der(22)t(9;22)[5] or +der(22)t(9;22)x2[12],+1~6mar[cp20].

The complete output (i.e. on- and off-target reads) from nanopore sequencing of DNA from the diagnostic sample was assessed for CNAs. In concordance with the diagnostic cytogenetic result, we detected +4, +6, +13, and +14. Additionally, nanopore sequencing showed evidence for +21, deletion of 9p, and partial duplications of chromosomes 2 and 8 that may correspond to the marker chromosomes detected by conventional karyotyping (Supplementary Fig. 4A). We did not detect a signal for +15. The remaining samples were also analysed for the presence of CNAs and an 843 kb deletion of chr4 in S_7 was detected (Table 3), representing the causative SV in the generation of *FIP1L1::PDGFRA* (Supplementary Fig. 4B). No other significant CNAs were observed in the remaining 18 samples.

## Discussion

We have shown that targeted nanopore sequencing using adaptive sampling can be effectively used for high-resolution detection of diverse TK-fusions in CML and MLN-TK. Long read sequencing is particularly suited to the detection of SVs [20] and has been previously utilised in the detection of translocations in other cancer types [31–34], however these approaches required either whole genome sequencing or targeting of genes of interest via PCR or CRISPR-Cas9 methods, increasing costs and complexity. The development of adaptive sampling allows for PCR-free, partner-agnostic detection and characterisation of translocations and gene fusions. Adaptive sampling has several advantages over other approaches: (i) it is fast (<72 h) and not constrained by batching requirements; (ii) bespoke sample preparation and DNA extraction is not required; (iii) high flexibility: it is trivial to include more ROIs if required by simply adding their genomic coordinates to the BED file containing the target definitions, avoiding potentially lengthy design and validations of other targeted approaches [35]; (iv) being highly targeted, it can be readily performed on MinION flow cells and is thus more economical and requires less computing capacity than long read WGS [34].

It is important to be aware of the limitations of any method when looking for potential drivers of disease. Our approach showed a persistent background signal of false-positive SVs supported by 1–2 sequence reads, consistent with the presence of artefactual chimeric reads arising from the ligation-based library preparation and/or fast sequential pore loading on the flow cell [36]. With an average sequencing depth of 36x, it is therefore unlikely that any true SVs with a variant allele frequency below 10–15% would be reliably detectable. This would likely not present a problem for identifying SVs in presentation samples with a relatively high clonal burden; however, it would be unsuitable for monitoring follow-up samples with reduced tumour load. We suggest an initial minimum threshold of 3 SV supporting reads to make a fusion call, although this could be lowered in specific cases where a potential SV has been identified involving a known TK and unknown partner. We also observed decreased coverage when strand information was defined in the target BED file (Fig. 2B, Supplementary Fig. 1). This behaviour was not documented in the ONT best practice guidelines at time of use but has been addressed in more recent revisions and is something users should be aware of to maximise sequence coverage.

We identified rearrangements arising from inter-chromosomal translocations (16/18 samples) as well as a deletion (S_7, *FIP1L1::PDGFRA*) and 26.9 Mb inversion (S_18) leading to *PRKG2::PDGFRA*. *PRKG2* has been previously reported as a *PDGFRB* fusion partner [37–39], however it has only been recently described as a partner to *PDGFRA* in two cases of myeloid neoplasia, including detection by optical genome mapping as part of a highly complex karyotype with an inversion of 4q [40, 41]. The benefit of base-pair resolution is highlighted by S_17, where FISH analysis was thought to have identified *ETV6::PDGFRA*. Nanopore sequencing showed there was no direct involvement of *PDGFRA*, with the breakpoint 149 Kb upstream of this gene. False positive *ETV6::PDGFRA* results from FISH analysis have been reported in cases of AML with t(4;12) that were unresponsive to imatinib, and were revealed to have no involvement of *PDGFRA* on subsequent sequencing analysis [42, 43]. To our knowledge, this is the first case of MLN-TK with similar findings.

As well as detecting known recurrent TK rearrangements, we also identified a previously undescribed *AGAP2::PDGFRB* fusion in patient S_14 (Fig. 5). *AGAP2* (Arf-GAP With GTPase, ANK Repeat and PH Domain-Containing Protein 2) is located on 12q14.1. It is thought to be involved in various cellular signalling pathways and is implicated in fibrosis in various organs [44]. The AGAP2 protein contains two ankyrin domains (Ank) at the C-terminal end which would be predicted to be retained in a fusion protein (Fig. 5D). Ank repeats are involved in protein-protein interactions, and have been shown to mediate dimerisation [45], thought to be necessary for activation of TK fusions [46].

Genomewide CNA analysis using the rejected, off-target reads is also a potentially useful aspect of adaptive sampling. We assessed a sample with a complex karyotype (S_2) and our results were broadly concordant with the diagnostic karyotype. We did not detect any support for a + 15 by nanopore sequencing, although we did detect evidence for a + 21 that was not present in the cytogenetic analysis, raising the possibility that this represents a mis-assigned +18 from the karyotype. We expect that prognostically important recurrent CNAs in myeloid neoplasms, such as -7/del7q [47], del5q [48], and trisomy 8 [49], would also be detectable by adaptive sampling, although this remains to be validated.

In conclusion, our approach was able to rapidly detect fusion genes at base pair resolution generated by different classes of SV, resolve complex variants and identify novel partner genes. We anticipate that nanopore sequencing will readily become part of the repertoire of approaches used for the diagnostic work up of patients with known or suspected haematological malignancies.

## Supplementary information

Supplementary Material

Supplementary Tables

## Data Availability

Sequence data is available from the authors on request
