# Supplementary material for: Long read nanopore DNA sequencing with adaptive sampling to identify tyrosine kinase fusion genes
Source: Leukemia. 2025 Nov 18;40(1):37–46. doi: 10.1038/s41375-025-02801-5 (PMC12789007; doi:10.1038/s41375-025-02801-5)
Supplement: Supplementary file 1 — Supplementary Material [file 41375_2025_2801_MOESM1_ESM.pdf]

Supplementary Material

Long read nanopore DNA sequencing with adaptive sampling to identify tyrosine kinase fusion genes

Matthew Salmon, Nicole Naumann, Jenny Rinke et al.

Supplementary Methods

Adaptive Sampling panel design

To ensure maximum coverage of the ROI is achieved, a buffer sequence is added to the end of each target locus. This ensures sequence reads that begin outside a target are not rejected prematurely (e.g. a 5kb long DNA fragment that begins 1kb upstream of a target should not be rejected, as it contains 4kb of ROI sequence). The length of the buffer sequence was experimentally determined to match the N90 of a sequence library (i.e. 90% of on-target reads containing ROI sequence should be accepted by AS) and was set at 11Kb. The total size of the targeted genes and buffer sequence was 25.32 Mb, representing 0.76% of the hg38 genome (approx. 3.3 Gb). The panel was designed with reference to the ONT adaptive sampling best practice guidelines (available via the ONT community website). A BED file describing the genomic coordinates of all targets plus buffer sequence was generated for use during adaptive sampling (Supplementary Table 1).

For 2 samples in the cohort (S_12 and S_18) we added additional targets to the BED file to improve the detection capability for suspected rearrangements. Either whole genes

were targeted as described in the main methods, or regions spanning a suspected breakpoint(s) in intergenic regions (Supplementary Table 4).

RT-qPCR MRD analysis

RT-qPCR was performed to assess the levels of *AGAP2::PDGFRB* transcript in 5 follow up cDNA samples from S_14 using the primer and probe sequences shown in supplementary table 5. Triplicate reactions were performed for each sample, and the ddCt method (1) was used to determine expression of the fusion transcript in follow samples, relative to the diagnostic sample. *ABL1* was selected as the control gene, using the Europe Against Cancer primer and probe set ENF1003, ENR1063, and ENPr1043 (2).

Supplementary Figures

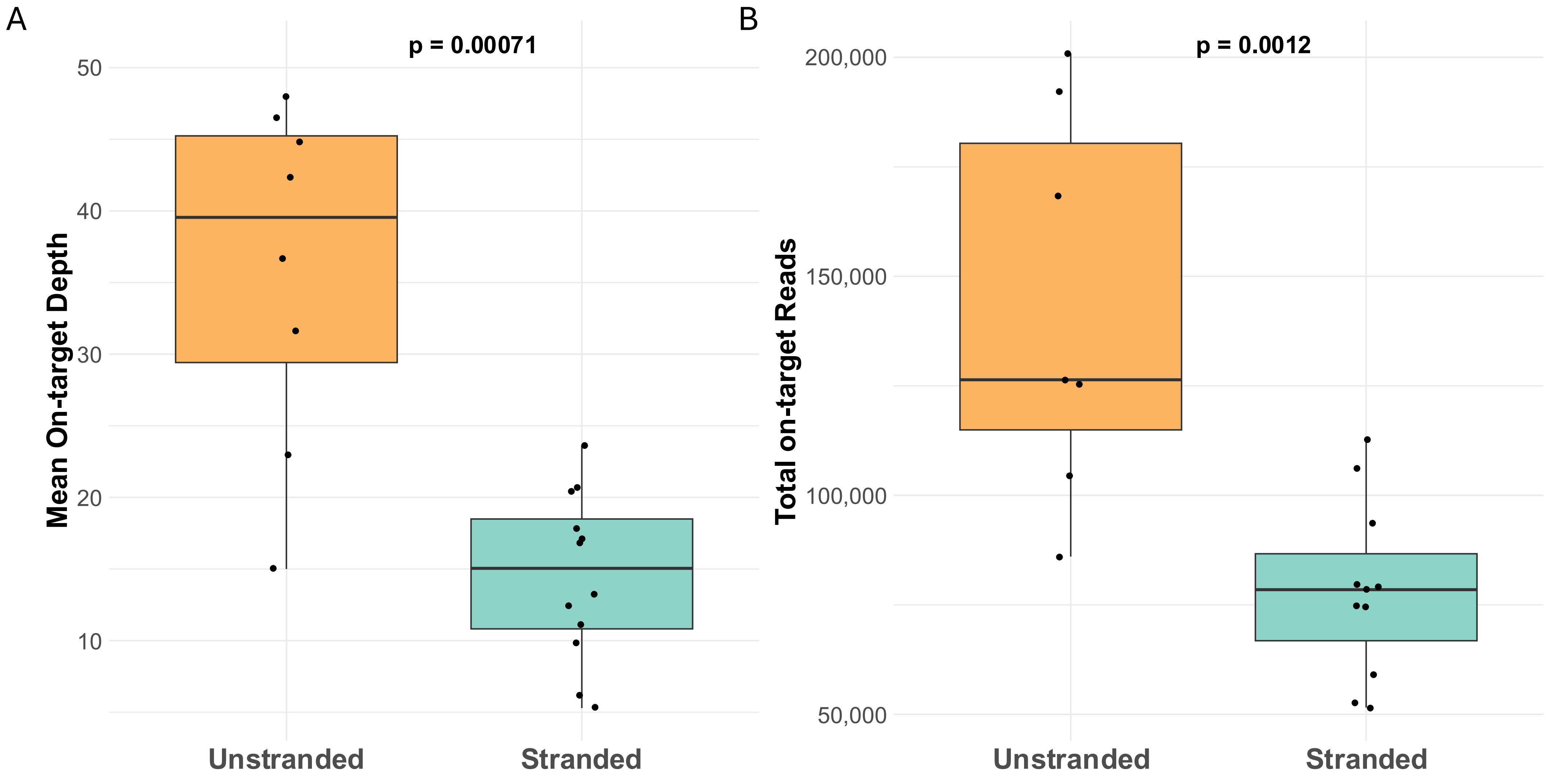

Supplementary Figure 1. Comparison of A) Mean on-target sequence depth and B) total number of on-target reads where an unstranded or stranded bed file was used. Wilcoxon rank sum test. S_11 and S_17 excluded from analysis of total on-target reads due to the targeting of addition loci.

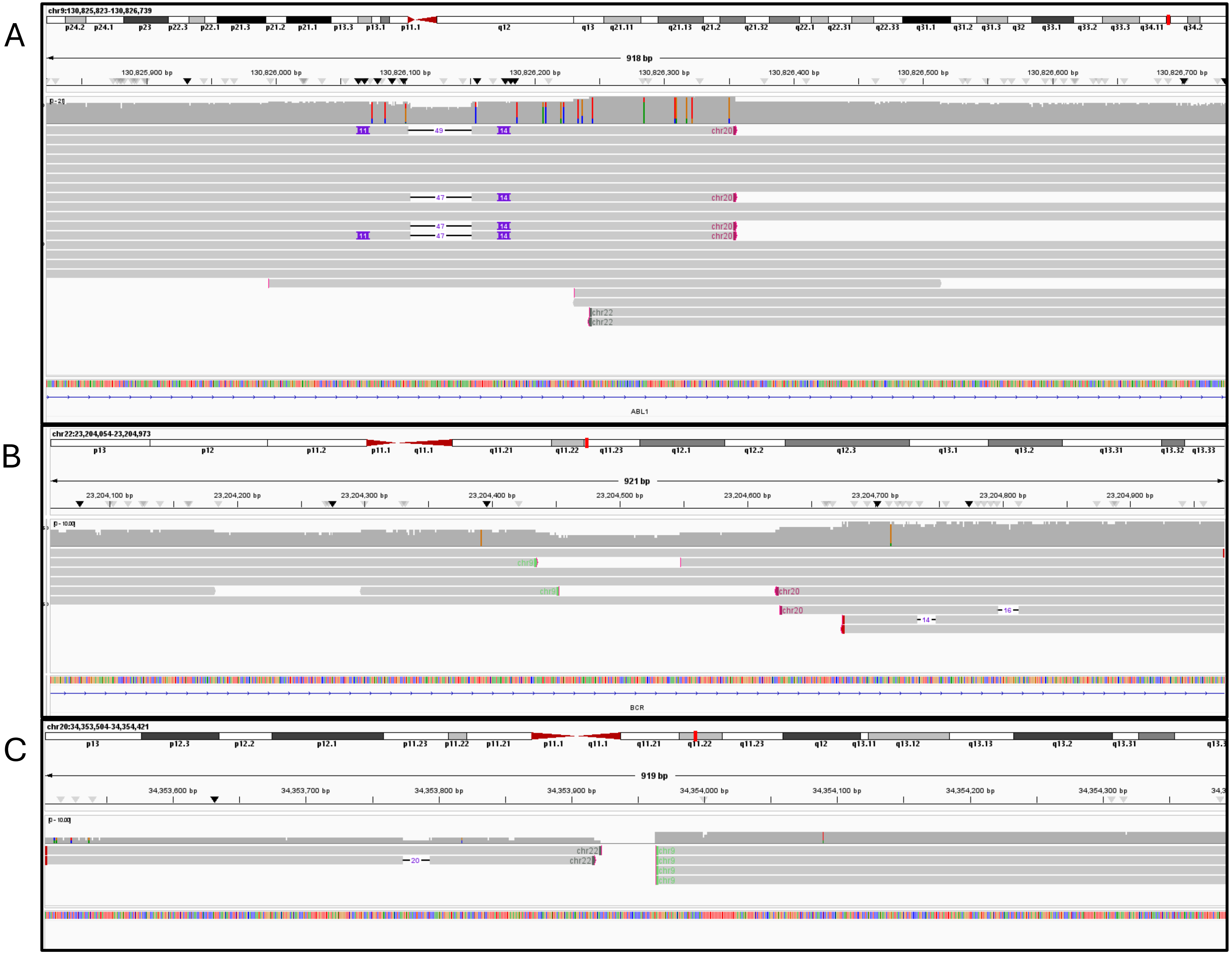

Supplementary Figure 2. IGV screenshots showing split reads spanning A) t(9;22), B) t(9;20), and C) t(20;22) rearrangements in S_5

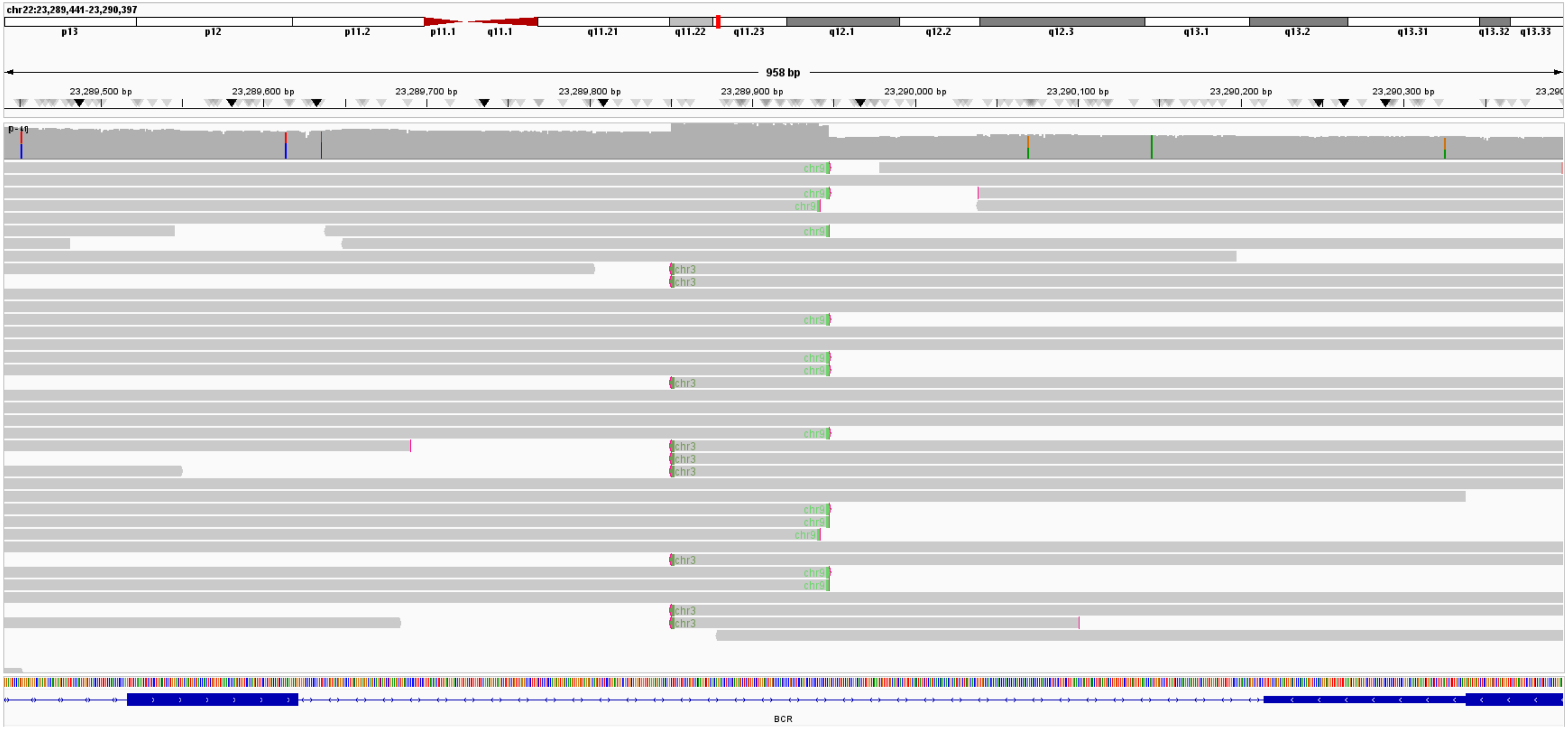

Supplementary Figure 3. IGV screenshot showing breakpoints in *BCR* for t(9;22) and t(3;22) in S_6. Partner chromosomes are indicated on fusion spanning reads.

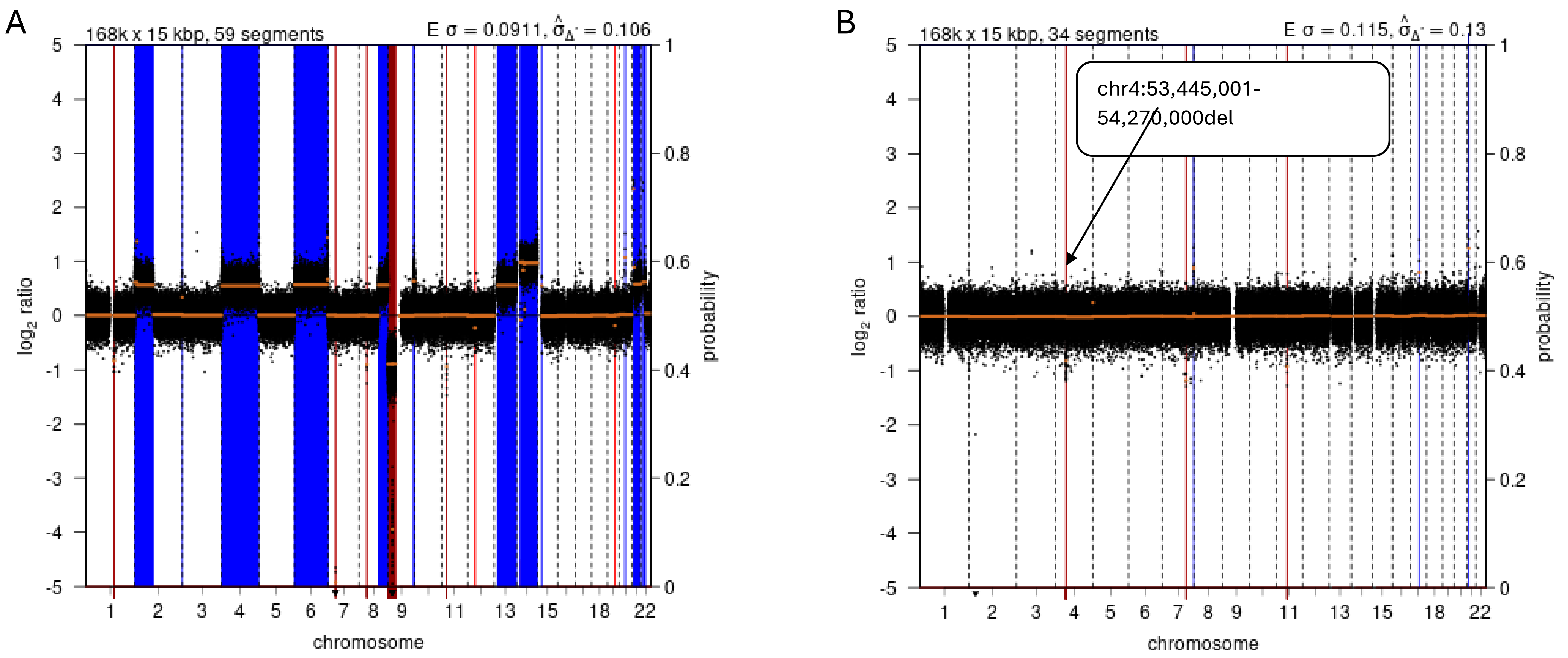

<u>Supplementary Figure 4.</u> CNAs called using QDNAseq showing A) the complex karyotype of S_2, and B) results from S_7 showing the deletion on chr4 covering the *FIP1L1::PDGFRA* fusion locus. Other gains/losses are of unknown significance. Copy number gains shown in blue, losses in red.

Supplementary references.

1. Livak KJ, Schmittgen TD. Analysis of relative gene expression data using real-time quantitative PCR and the 2(-Delta Delta C(T)) Method. Methods. 2001;25(4):402-8.
2. Beillard E, Pallisgaard N, van der Velden VH, Bi W, Dee R, van der Schoot E, et al. Evaluation of candidate control genes for diagnosis and residual disease detection in leukemic patients using 'real-time' quantitative reverse-transcriptase polymerase chain reaction (RQ-PCR) - a Europe against cancer program. Leukemia. 2003;17(12):2474-86.
